# Fabrication and Evaluation of PCL/PLGA/β-TCP Spiral-Structured Scaffolds for Bone Tissue Engineering

**DOI:** 10.3390/bioengineering11070732

**Published:** 2024-07-19

**Authors:** Weiwei Wang, Xiaqing Zhou, Haoyu Wang, Gan Zhou, Xiaojun Yu

**Affiliations:** 1 Department of Biomedical Engineering, Charles V. Schaefer School of Engineering and Sciences, Stevens Institute of Technology, Hoboken, NJ 07030, USA; wwang19@stevens.edu (W.W.); xzhou12@stevens.edu (X.Z.); hwang40@stevens.edu (H.W.); 2 Department of Chemistry and Chemical Biology, Charles V. Schaefer School of Engineering and Sciences, Stevens Institute of Technology, Hoboken, NJ 07030, USA

**Keywords:** spiral scaffold, bone regeneration, polymer, β-tricalcium phosphate

## Abstract

Natural bone is a complex material that has been carefully designed. To prepare a successful bone substitute, two challenging conditions need to be met: biocompatible and bioactive materials for cell proliferation and differentiation, and appropriate mechanical stability after implantation. Therefore, a hybrid Poly ε-caprolactone/Poly(lactic-co-glycolide)/β-tricalcium phosphate (PCL/PLGA/β-TCP) scaffold has been introduced as a suitable composition that satisfies the above two conditions. The blended PCL and PLGA can improve the scaffold’s mechanical properties and biocompatibility compared to single PCL or PLGA scaffolds. In addition, the incorporated β-TCP increases the mechanical strength and osteogenic potential of PCL/PLGA scaffolds, while the polymer improves the mechanical stability of ceramic scaffolds. The PCL/PLGA/β-TCP scaffold is designed using spiral structures to provide a much better transport system through the gaps between spiral walls than conventional cylindrical scaffolds. Human fetal osteoblasts (hFOBs) were cultured on spiral PCL/PLGA/β-TCP (PPBS), cylindrical PCL/PLGA/β-TCP (PPBC), and cylindrical PCL scaffolds for a total of 28 days. The cell proliferation, viability, and osteogenic differentiation capabilities were analyzed. Compared with PCL and PPBC scaffolds, the PPBS scaffold exhibits great biocompatibility and potential to stimulate cell proliferation and differentiation and, therefore, can serve as a bone substitute for bone tissue regeneration.

## 1. Introduction

Due to the high incidence of bone defects, there is a huge clinical demand for bone grafts. Bone defects are mainly caused by trauma, bone tumors, congenital deformation, and bone infections [1]. Depending on the donor tissues and materials, bone grafts can be classified as autograft, allograft, or xenograft [2,3]. Autologous bone graft is considered to be the most ideal bone graft for repairing bone defects because of its high efficiency, excellent osteoconduction, and mechanical properties. However, autograft treatment has shown weaknesses such as donor site morbidity, additional surgery, and limited availability [4,5]. In order to overcome the shortcomings of autograft, allograft and xenograft materials have been developed. However, both of them not only produce immunogenic responses, but also have a high failure rate due to low osteogenicity and osteoinductivity [6,7]. As a result, many efforts have been dedicated to the development of bone tissue regeneration that can circumvent many of the shortcomings mentioned above.

Three-dimensional (3D) polymeric scaffolds with excellent biocompatibility and mechanical properties have recently been introduced as bone grafts for repairing and restoring a variety of large bone defects [8,9]. The polymers used to prepare these 3D polymeric scaffolds can be natural or synthetic, biodegradable or non-biodegradable. Among these polymers, Poly ε-caprolactone (PCL), a member of the synthetic and biodegradable polymers, has been widely used in bone tissue regeneration because of its good biocompatibility [10,11]. In addition, the melting temperature and glass transition temperature for PCL are 59–64 °C and −60 °C, respectively. Therefore, PCL has high toughness and excellent mechanical properties at room temperature [12]. Recently, Fuchs et al. [13] made PCL scaffolds using a melt electrospinning writing method for oral bone regeneration. However, PCL scaffolds have their own limitations as bone defect implants due to their extreme hydrophobicity and lack of surface biological activity. Poly(lactic-co-glycolide) (PLGA), a combination of poly(lactic acid) (PLA) and poly(glycolic acid) (PGA), is another biocompatible and biodegradable polymer, widely used in tissue engineering scaffolds [14]. Compared with PCL, PLGA shows better cell adhesion because of its hydrophilic property [15]. Morgan et al. [16] used the wet-spinning method to successfully prepare PLGA scaffolds with human bone marrow stromal cells for natural bone repair and regeneration. However, this scaffold exhibited relatively low mechanical strength. To overcome the above problems, a scaffold constructed with blended PCL and PLGA has been used as an advantageous composition for bone regeneration because of its improved mechanical properties and biocompatibility compared with single PCL or PLGA scaffolds [17]. Moreover, PCL exhibits a longer degradation time than PLGA. Therefore, the volume occupied by PLGA can form in situ pores on the scaffold at the later stage of implantation, and the porous structure can promote cell proliferation and vascularization. In order to further optimize the biocompatibility and osteoconduction of the PCL/PLGA scaffold, bioceramic components, such as hydroxyapatite (HA) or β-tricalcium phosphate (β-TCP), can be incorporated into the polymeric scaffold [18,19,20]. β-TCP is the main inorganic component of natural bone. Because of its excellent bone conductivity and strong binding ability with the host bone tissue, β-TCP is extremely potent in stimulating bone formation [21,22]. It has been studied and widely used for bone regeneration for decades. The earliest studies and clinical applications of β-TCP were performed before 1980 [23]. It has been one of the most attractive bone graft substitute materials as it is osteoconductive and osteoinductive. The greatest advantage of using β-TCP in bone regeneration is that this material is resorbable and can be replaced by new bone easily [24,25]. Although as a ceramic, β-TCP has high mechanical strength but is brittle, the combination of β-TCP and some other synthetic polymers can efficiently improve the mechanical properties of scaffolds. Several studies have shown that β-TCP-containing biodegradable polymer composites can be used for bone tissue regeneration. For example, Kikuchi et al. [26] successfully developed biodegradable composites consisting of β-TCP and PLGC (poly (l-lactide-co-glycolide-co-ε-caprolactone)) using a heat-kneading method for good regeneration of beagles’ mandibular bones. Lam et al. [27] fabricated novel composite blends of poly(L-lactide-co-D, L-lactide)/β-TCP by an extrusion deposition technique exhibiting good biocompatibility and mechanical stress.

**Scheme 1:**
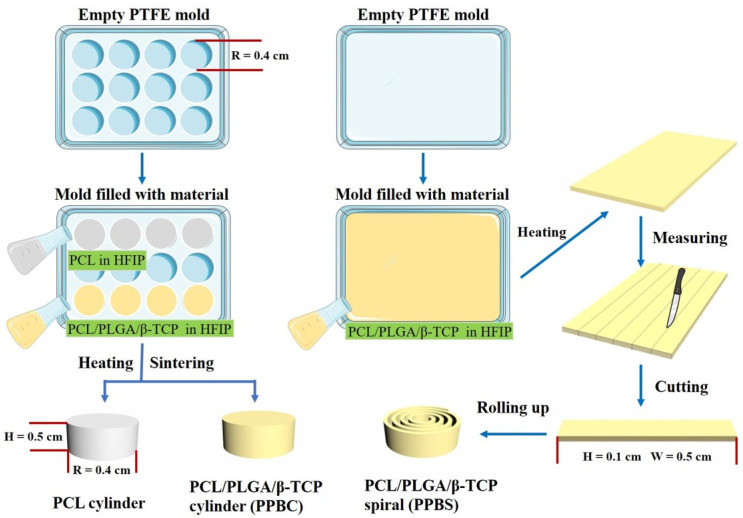
Schematic diagram of the step-by-step preparation of different scaffolds.

Therefore, our group developed a blended PCL/PLGA/β-TCP scaffold for bone tissue regeneration. The scaffold, with a different weight ratio of PCL and PLGA, will exhibit quite different characteristics such as mechanical strength and degradation rate. Based on our previous results [28], three scaffolds with different weight percent ratios were prepared for the study: 20PCL/60PLGA/20βTCP, 40PCL/40PLGA/20βTCP, and 60PCL/20PLGA/20βTCP. The results indicated that, compared with the other two, 40PCL/40PLGA/20βTCP (PPBC) was characterized by a higher percentage of porosity and better mechanical strength over time. Thus, PPBC was selected for further study. In addition to the composition of the scaffold, our group has designed a spiral-structured scaffold (see Scheme 1) that mimics the natural bone structure and provides open gaps and sufficient space for cell attachment, migration, proliferation, and differentiation during in vitro culture [29,30]. The spiral-structured scaffold our group designed can provide thinner scaffold walls for cells to easily grow across. Compared with conventional cylindrical scaffolds, the gaps between scaffold walls also provide a much better transport system to ensure sufficient nutrient supply and metabolic waste removal [31]. Compared with the traditional porous scaffolds with interconnected porosity, our design leaves more space for tissue growth and decreases the amount of material needed, therefore decreasing the risk of possible chronic inflammation caused by the degradation of some polymers. In this study, PCL/PLGA/βTCP were prepared in both cylindrical and spiral structures, and the cylindrical PCL scaffold was prepared as the control. The morphology, mechanical strength, and degradation of each scaffold was characterized. Moreover, in order to investigate their in vitro osteogenesis capabilities, the cellular responses of human fetal osteoblasts (hFOBs) to the scaffolds were studied, and the results indicate that spiral PCL/PLGA/βTCP is a suitable composition for bone graft substitution.

## 2. Materials and Methods

### 2.1. Materials

For this study, 3-(4,5-dimethylthiazol-2-yl)-5-(3-carboxymethoxyphenyl)-2-(4-sulfophenyl)-2H-tetrazolium) (MTS) assay kit, Cell TiterBlue assay kit, and alkaline phosphatase (ALP) assay kit were obtained from Promega (Madison, WI, USA) and Abcam (Cambridge, MA, USA), respectively. Alizarin red S (ARS) staining solution (40 mM) and cetylpyridinium chloride (CPC) were purchased from Sigma-Aldrich Company (St. Louis, MO, USA). Cell lines and culture-related products were acquired from Invitrogen (Carlsbad, CA, USA). All other chemicals were purchased from Sigma-Aldrich Company (St. Louis, MO, USA) and used as received (analytical grade). Deionized water (D.I. water) and 0.10 M phosphate buffer saline (PBS) were used for solution preparation.

### 2.2. Scaffold Fabrication

We have previously established methods to fabricate cylinder and spiral scaffolds. Briefly, the cylinder scaffolds were prepared by dissolving the materials—PCL, or a mixture of PCL, PLGA, and βTCP—in 1,1,1,3,3,3-Hexafluoro-2-propanol (HFIP), and then heating the solution to 90 °C for 30 min in the premade PTFE mold with a radius of 4.0 mm, followed by compression from the top with a pressure level of 80,000 Pa using a punch. The scaffolds were left in the chemical hood overnight to evaporate all the HFIP. The final scaffolds were trimmed to achieve a height of 5.0 mm. As for the spiral scaffolds, they were prepared in three steps. First, the material solution was poured into a flat-bottom PTFE mold with a heating temperature of around 70 °C. Next, the mold was gently shaken to ensure that the solution was evenly distributed. After that, the mold with the solution was left in the chemical hood for 3 min to evaporate most of the HFIP, to achieve a thin layer of film with a thickness of around 0.3 mm. Finally, the resulting film was cut into several strips with a width of 5.0 mm and rolled up into a spiral shape. The final radius of each spiral scaffold was the same as that of the cylinder scaffolds, which is 4.0 mm (see Figure S1).

### 2.3. Water Contact Angle (WCA)

The WCA of the scaffolds was measured by a standard Ramé-Hart advanced goniometer model 500-F1 (Netcong, NJ, USA) at room temperature and 70% relative humidity (R.H.). Briefly, a 10 μL drop of water was carefully placed on each scaffold surface. Then, the WCA values and images were recorded and captured by the DROP image advanced software. Three samples of each scaffold were measured to calculate the average value.

### 2.4. Attenuated Total Reflectance-Fourier Transform Infrared Spectroscopy (ATR-FTIR)

ATR-FTIR spectra of all three scaffolds were measured with the JASCO FT/IR-460 Spectrometer (Easton, MA, USA), and Opus software was used to collect and analyze the data. Specifically, the scaffold was first placed on the ATR crystal platform, then the top screw was gently rotated to fix the solid scaffold and finally start the measurements.

### 2.5. Mechanical Properties Test

The mechanical properties of different samples were tested by the Instron Model 5965 Material Test System. Briefly, disc-shaped samples (approximately 10 mm in diameter and 5 mm in height) were compressed at a rate of 250 µm/s until the compressing load reached the maximum of 4500 N. The moduli of different samples were calculated based on the resulting linear part of the stress–strain curves.

### 2.6. Scaffold Degradation and Porosity

A scanning electron microscope (SEM) was used to observe the degradation of the scaffolds. To be specific, the scaffolds were separately immersed in 1 mL of PBS for a total of 28 days. At specific time points (day 14, 21, and 28), the scaffolds were removed from the PBS and air-dried. Later, the scaffolds were sputter-coated with a 1.5 nm thick Au layer and images were taken under an Auriga Modular Cross Beam workstation (Carl Zeiss, Inc., Oberkochen, Germany) at 2.00 kV. In order to quantify the degree of degradation, the porosity of the scaffolds was measured. This is because PLGA biodegrades faster than PCL, so the volume occupied by PLGA will change into a porous network over time. Specifically, the porosity of the scaffolds (n = 5) was calculated according to Archimedes’ principle equation [32]:

Porosity = (W_sat_ − W_dry_)/(W_sat_ − W_sus_) × 100% (1)

where W_sat_ and W_sus_ represent the weight of a scaffold when saturated and when suspended with water, respectively. W_dry_ is the dry weight of the scaffold.

### 2.7. Cell Culture and Seeding

The human fetal osteoblast (hFOB) cell line was maintained in a 1:1 mixture of Ham’s F12 Medium and Dulbecco’s Modified Eagle’s Medium, supplemented with 2.5 mM L-glutamine, 1% antibiotics (penicillin/streptomycin), and 10% fetal bovine serum (FBS). The cells were cultured at 37 °C in a 5% CO_2_ incubator, and the medium was changed every 2 days. Prior to cell seeding, the scaffolds were sterilized by soaking in 70% ethanol for 2 h and then washed three times with sterile PBS. During cell seeding, 50 μL of cell suspension containing 1 × 10^5^ hFOB cells was gently and evenly added onto each scaffold in a 48-well tissue culture plate. The cells were allowed to attach for 2 h before flooding with 1 mL culture medium per well. The cultures were maintained for a total of 28 days, and the medium was changed every 2 days before the 14th day, and every day after the 14th day.

### 2.8. Cell Proliferation Test

MTS assay was used to evaluate the proliferation of hFOB cells on different scaffolds. At the end of each pre-designated time point (day 1, 7, 14, 21, and 28), the scaffolds (n = 5) were rinsed twice with cold PBS. Then, 200 μL of MTS reagent was added onto the scaffold along with 500 μL of medium and incubated at 37 °C for 2 h. Afterward, 200 μL of solution of each sample was transferred to a 96-well plate, and the absorbance of the solution was measured at 490 nm by using a UV–vis microplate reader (BioTek Instruments, Inc., Winooski, VT, USA).

### 2.9. ALP Assay

At pre-designated time intervals of 14, 21, and 28 days, the osteoblast phenotype differentiation was measured by using the ALP assay kit according to the manufacturer’s instructions. In detail, the scaffolds were taken out and washed twice with PBS. Each scaffold (n = 3) was then transferred to a 1.5 mL Eppendorf tube containing 1 mL of assay buffer. The samples were centrifuged at 15,000 rpm/min for 15 min at 4 °C to remove any insoluble materials. Next, the supernatant was collected and transferred to a new Eppendorf tube. Then, 50 μL of 5 mM *p*NPP solution was added to each well (96-well plate) containing 80 μL of the above supernatant. Afterward, the 96-well plate was incubated at room temperature for about 60 min in the dark, and subsequently, 20 μL of stop solution was added to stop the reaction. At the same time, a fresh set of standards also needed to be prepared. Specifically, 10 μL of the ALP enzyme solution was added to 120 μL of each *p*NPP standard well and incubated for 1 h, followed by adding 20 μL of stop solution. The absorbance was measured at 405 nm using a UV–vis microplate reader, and the ALP activity (U/mL) was calculated as follows:

(2) $$\mathrm{ALP}\;\mathrm{activity}=\frac{Amount\;of\;pNP}{Reaction\;time\times Original\;sample\;volume}\times Sample\;dilution\;factor$$

where the amount of *p*NP was calculated according to the standard trend line equation, the reaction time was set at 60 min, the original sample volume was 80 μL, and the dilution factor was 1.

### 2.10. ARS Staining and Quantification

The mineralized matrix deposition of the hFOB cells on the scaffolds was assessed by ARS staining, which quantified the calcium deposition. To be specific, the scaffolds (n = 3) were fixed in 4% paraformaldehyde (PFA) for 30 min and washed with PBS three times on day 14, 21, and 28. Then, the scaffolds were stained with 1 mL ARS solution (40 mM) for 30 min under gentle shaking. Afterward, the scaffolds were washed five times with D.I. H_2_O to remove excess ARS, after which the optical images were taken (Nikon SMZ1500, Melville, NY, USA). For ARS quantification, the scaffolds were soaked in 1 mL 10% (*w*/*v*) CPC 10 mM sodium phosphate solution overnight at room temperature to extract the stained ARS. The absorbance was measured at 562 nm using a UV–vis microplate reader, and the standard curve was prepared by dissolving ARS in CPC with different concentrations.

### 2.11. Cell Adhesion on Scaffolds

Fluorescent images allow us to directly observe the cells’ growth and attachment to the scaffolds. Briefly, after 14, 21, and 28 days of incubation, the scaffolds were washed with cold PBS three times, fixed in 1 mL 4% formaldehyde solution for 45 min, and permeabilized in 1 mL 0.1% Triton X-100 for 30 min at room temperature. The scaffolds were then blocked in 1 mL solution containing 3% BSA for 45 min. Next, the scaffolds were immersed in Phalloidin (Alexa Fluor 488,Biotium, Fremont, CA, USA) solution for 1 h to label the F-actin, and then the scaffolds were rinsed with PBS, then stained for 10 min with 4′,6-diamidino-2-phenylindole (DAPI) to label the nuclei. Finally, the scaffolds were visualized under a Nikon Eclipse 80i epifluorescence microscope.

### 2.12. Statistical Analysis

All the data above were presented as mean ± standard deviation and evaluated by the one-way ANOVA test. The results were considered statistically significant if they had a *p*-value of less than 0.05.

## 3. Results and Discussion

### 3.1. Hybrid Scaffold Characterization

**Figure 1:**
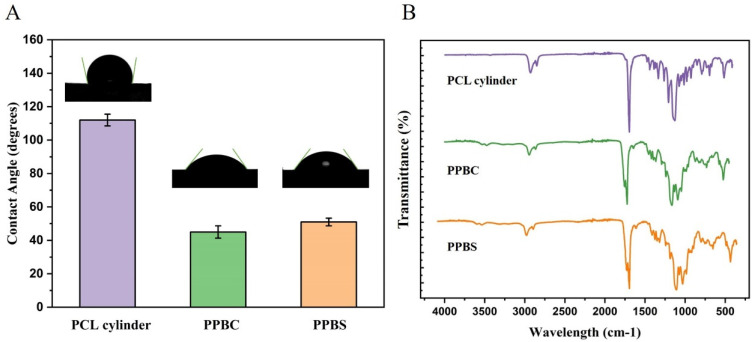
(**A**) Water contact angles and (**B**) ATR-FTIR analysis of the PCL cylinder, PPBC, and PPBS scaffolds.

PCL is a U.S. Food and Drug Administration (FDA)-approved biodegradable and biocompatible polymer, but its application is limited because of its hydrophobicity. Therefore, PCL is often blended with other polymers to increase its hydrophilic property. PLGA is a biodegradable, non-toxic, and hydrophilic polymer, and it can form micropores on its surface to improve nutrient permeability. β-TCP is not only osteoconductive, but also osteoinductive. Therefore, we designed a hybrid material of PCL, PLGA, and β-TCP for use as a potential bone graft material. From Figure 1B we can see that both PPBC and PPBS show fingerprint peaks of PLGA (the region of 1750 cm^−1^, the stretching vibration of the carbonyl groups (C=O)) and β-TCP (two sharp peaks at 604^−1^ and 544 cm^−1^), indicating that we had successfully mixed the three materials and they did not undergo phase separation.

As shown in Figure 1A, the contact angles for the PCL, porous cylinder, and porous spiral scaffolds were 111.5°, 47.1°, and 51.7°, respectively. These results indicate that the hybrid of PCL, PLGA, and β-TCP greatly improved the material’s hydrophilicity, which could further improve the cell affiliation to the scaffold.

**Figure 2:**
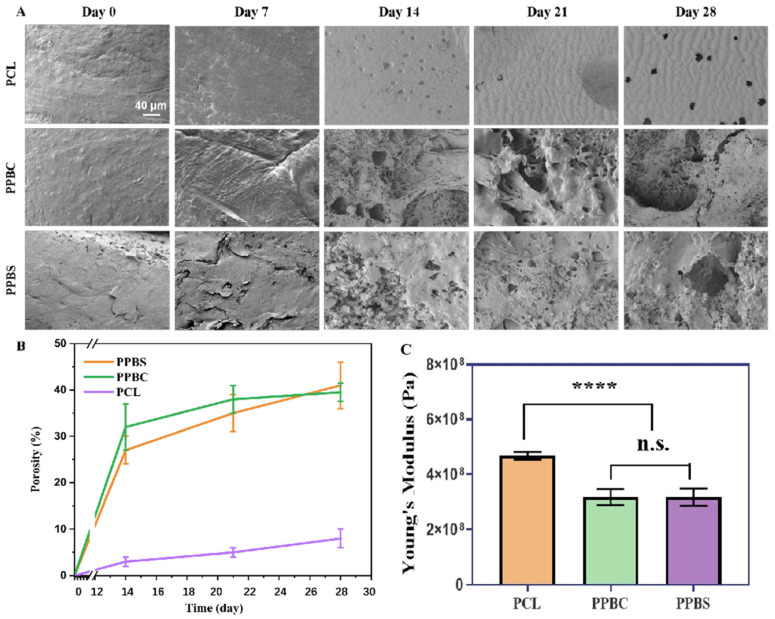
(**A**) SEM images of the morphology of PCL (top), PPBC (middle), and PPBS (bottom) in culture mediums after different degradation times (scale bar = 40 μm); (**B**) the porosity of PPBS, PPBC, and PCL after 0, 7, 14, 21, and 28 days; (**C**) the tested Young’s moduli of the three types of scaffolds (****: *p* < 0.0001; n.s.: *p* > 0.05 no significant difference. n = 5.).

The degradation of 50/50 PLGA plays an important role in inducing cell growth. PCL is a polymer with a relatively low degradation rate in physiological conditions, while PLGA degrades faster than PCL. Therefore, over time, the volume occupied by PLGA in the scaffold would be transformed into a porous network, which could enable cell infiltration and allow blood vessels to grow inside. The surface morphology of each scaffold was analyzed by SEM during degradation in PBS, as seen in Figure 2A. After degradation for up to 28 days, there was no obvious change in the structure of the PCL scaffold. In contrast, the surface morphology of both the PPBS and PPBC scaffolds began to degrade to form small pores after 14 days of incubation, and the pore sizes and pore interconnectivity also increased along with the degradation time, indicating that the PLGA was gradually disappearing. Later, in order to quantify the degree of degradation, the porosity of each scaffold was measured by using Archimedes’ principle equation. As shown in Figure 2B, we could see that the porosity of the PCL scaffold was approximately 3% after 14 days of incubation and only around 8% at the end of the experiment. However, the porosity of PPBS and PPBC increased from around 30% on day 14 to about 40% after 28 days of culture, which was 5 times higher than that of PCL. The quantitative analysis results were consistent with the SEM images and illustrated the progressive degradation of PLGA.

Mechanical properties, especially Young’s modulus, comprise one of the key parameters for designing a bone graft. From Figure 2C we can see that the PCL cylinder scaffold has an average Young’s modulus of 468.3 MPa, whereas the average Young’s moduli of PPBC and PPBS are 317.6 MPa and 299.4 MPa, respectively. Although the PCL scaffold’s Young’s modulus is significantly larger than the PPBC and PPBS scaffolds’, the Young’s moduli of PPBC and PPBS are sufficient for bone graft materials. Also, the behaviors of cells on these scaffolds should be taken into consideration and are equally important as mechanical properties. As discussed above, the porous structure created by PLGA degradation would induce much better cell attachment and nutrient infiltration, and β-TCP would facilitate osteogenesis as well.

### 3.2. hFOB Proliferation on Scaffolds

**Figure 3:**
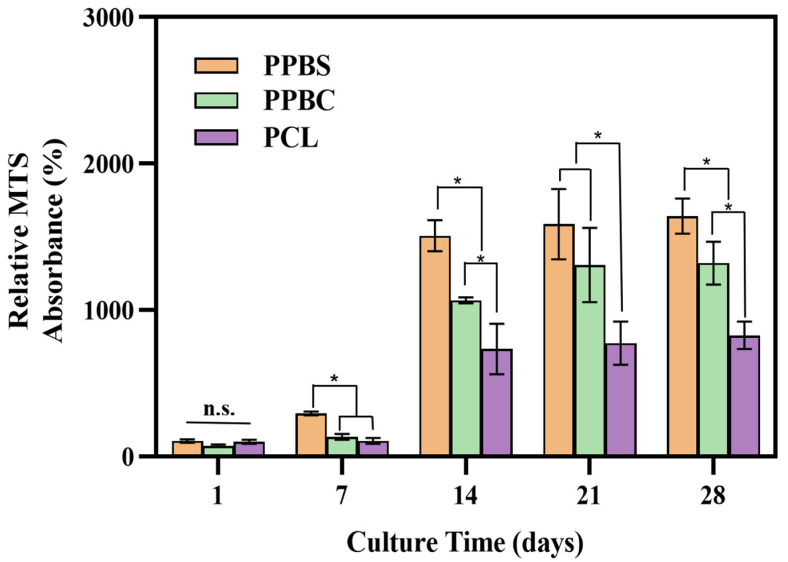
MTS assay showing hFOB cells’ attachment and proliferation on PPBS, PPBC, and PCL after 1, 7, 14, 21, and 28 days (*: *p* < 0.05; n.s.: *p* > 0.05 no significant difference. n = 5).

hFOB cells were seeded on the PCL, 50PCL/50PLGA/20β-TCP cylinder (PPBC), and 50PCL/50PLGA/20β-TCP spiral (PPBS) scaffolds, separately. As mentioned previously, the hybrid of PCL and PLGA can overcome the disadvantages of single use and show excellent cell proliferation and tissue formation. Moreover, a spiral-structured scaffold (such as PPBS) shows superiority to traditional scaffolds due to its open architecture, increased surface-to-volume ratio, and thinner scaffold wall, resulting in better medium inflow and deeper cell penetration into the scaffold. Considering the components and structures of these three scaffolds, we believed that compared to the pure PCL scaffold, the combined use of PCL/PLGA/β-TCP should allow better cell adhesion, and the spiral structure would have the most cell growth. In order to prove our hypothesis, hFOB cell proliferation on the PCL, PPBC, and PPBS scaffolds was quantitatively evaluated through MTS assay over a period of 28 days. As shown in Figure 3, the cell growth on all three scaffolds increased with culture time. However, PPBS demonstrated a much higher number of viable cells at all time points compared to PPBC and PCL. To be specific, the proliferation of hFOB cells on PCL, PPBC, and PPBS was not significantly different until day 7, when the number of attached cells on PPBS was approximately 2.8 times higher than that of PCL and PPBC. At day 14, the cell attachment on both PPBS and PPBC was significantly higher than the control PCL; however, PPBS showed greater proliferation than PPBC. At the end of 21 and 28 days, the cell numbers on all these scaffolds exhibited a small amount of growth, but PPBS showed the highest cell proliferation and attachment, with an absorbance value of 1.2 ± 0.1. Overall, PCL combined with PLGA and β-TCP in a spiral shape exhibited significantly enhanced cell growth at all time points of this study.

### 3.3. hFOB ALP Activity

**Figure 4:**
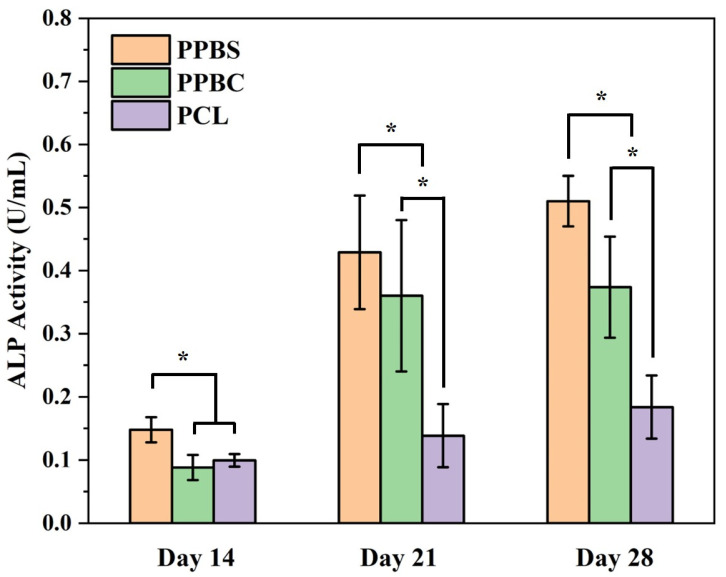
Alkaline phosphatase (ALP) activity of hFOB cells cultured on PPBS, PPBC, and PCL for 14, 21, and 28 days (*: *p* < 0.05. n = 5.).

The early matrix deposition and maturation of the hFOB cells on the scaffolds were evaluated by measuring the alkaline phosphatase (ALP) activity [33]. As shown in Figure 4, no significant ALP activity difference was observed between the cells cultured on PCL and PPBC scaffolds on day 14. However, on day 14, the ALP activity showed a significant increase in the PPBS scaffold compared to the other two scaffolds. In addition, after 21 days of incubation, the hFOB cells cultured on the PPBC and PPBS scaffolds exhibited enhanced ALP activity compared with those on the PCL scaffold. It was also observed that from day 21 to day 28, the ALP activity levels of the hFOB cells grown on the PCL, PPBC, and PPBS scaffolds did not increase a lot, but maintained similar differences, indicating that most of the osteoblast differentiation and bone matrix formation was completed on day 21. To summarize, the above results suggest that the combination of PCL, PLGA, and β-TCP could induce more cell growth and differentiation during the 28-day culture period compared with pure PCL. In addition to PLGA, we believe that β-TCP also played an important role in inducing osteogenic differentiation. β-TCP, which has good biocompatibility, biodegradability, and osteoconductivity, has been widely used as a bone substitute as it releases calcium ions, increasing the mechanical strength and osteogenic potential of scaffolds. Moreover, PPBS with a spiral structure showed the highest amount of ALP activity, which further indicates its novel scaffold design. The percentage of β-TCP (20%) used in this study was the maximum ratio that can be applied in hybrid materials without causing phase separation during scaffold fabrication. There would be better cell proliferation and more ALP activity if a higher percentage of β-TCP could be included in the hybrid material due to improvement of the fabrication method. This would be an interesting topic to investigate. Since the structure can also change the mechanical properties of composite materials, the influence of the weight percentage of β-TCP on different materials’ structures would be worthy of investigation.

### 3.4. ARS Staining and Quantification

**Figure 5:**
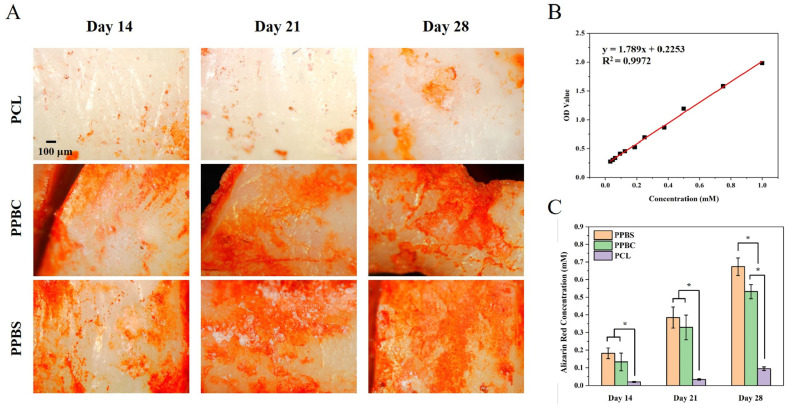
(**A**) Assessment of mineralization by Alizarin red S (ARS) staining of the cell-seeded PCL, PPBC, and PPBS at 14, 21, and 28 days; (**B**) the standard curve of ARS in cetylpyridinium chloride for quantification; (**C**) calcium deposition, quantified by ARS assay, extracted from days 14, 21, and 28 (*: *p* < 0.05. n = 5.).

We further investigated the osteogenic differentiation of hFOB cells on the PCL, PPBC, and PPBS scaffolds through assessment with the Alizarin red S (ARS) staining method, which is used to track the mineralization process in osteoblast cultures [34,35]. On day 14 (Figure 5A), the red staining covered nearly half of the entire surface area of the PPBC and PPBS scaffolds, indicating calcium deposition. In contrast, PCL did not show many red-stained areas. At the end of day 21, the amount of deposited calcium increased on the PPBC and PPBS scaffolds, both of which had a larger red area compared with that of the PCL scaffold. After 28 days of incubation, full red staining was observed on the PPBS scaffold, indicating the most mineral deposits. To quantify the ARS staining, the stained scaffolds were immersed in cetylpyridinium chloride and the dye was extracted, as previously described. The analysis of the quantification results (Figure 5C) was consistent with the staining images. To be specific, the absorbance values of the ARS deposited on PCL were 0.023 ± 0.001 on day 14, 0.034 ± 0.004 on day 21, and 0.095 ± 0.012 on day 28. However, the absorbance values for PPBS and PPBC increased significantly, from 0.182 ± 0.032 and 0.133 ± 0.053 on the 14th day to 0.673 ± 0.056 and 0.532 ± 0.041 on the 28th day. Taken together, both the staining images and quantification results show that the PPBS scaffold exhibited the highest ARS staining, followed by the PPBC scaffold, and finally the PCL scaffold. These results demonstrate that calcium deposition was dramatically increased for the combination of PCL, PLGA, and β-TCP, as compared to pure PCL. Additionally, significant differences were also observed between the PPBS and PPBC scaffolds, proving that in addition to scaffold composition, the spiral structure (PPBS) was more conducive to calcium deposition than the cylindrical structure (PPBC).

### 3.5. Fluorescent Images of hFOB on Scaffolds

**Figure 6:**
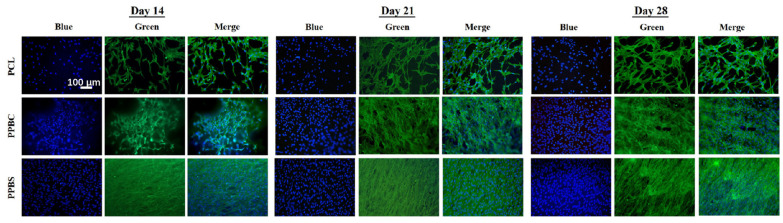
Fluorescent images of hFOB cells cultured on PCL, PPBC, and PPBS for 14, 21, and 28 days. Cells were stained by DAPI (blue) and Phalloidin (green).

The previous MTS results showed the hFOB cell proliferation and viability on PCL, PPBC, and PPBS scaffolds for a total of 28 days. Apart from the cell viability, in order to further observe the cell shape, membrane integrity, cell adhesion, and cell spreading [36], hFOB cells were cultured on three scaffolds for 14, 21, and 28 days, prior to which green (Phalloidin) and blue (DAPI) fluorescent probes labeled the F-actin and nuclei of the cells, respectively, followed by fluorescence microscopy. As shown in Figure 6, the pure PCL scaffold supported a certain degree of cell attachment and survival at all the time points studied, but this amount was significantly lower than that of the combination of PCL/PLGA/β-TCP. The hFOB cells cultured on the composite scaffolds PPBS and PPBC displayed good morphology and a dense layer cover during the course of the observation, indicating cell survival and propagation. In addition, the fluorescent images show that more cell growth was observed on the PPBS scaffold compared to the PPBC scaffold, especially on day 14. These results are consistent with the MTS results, and the potential reasons for the beneficial effects of PPBS in improving cell attachment and proliferation include its composition and unique structure. Moreover, it was observed that the growth and proliferation of the hFOB cells reached confluence on the PPBS scaffolds after 14 days of incubation, which was also in accordance with the MTS results from days 14 to 28.

## 4. Conclusions

In summary, we have successfully developed and prepared a 50PCL/50PLGA/β-TCP (weight ratio) scaffold with both cylindrical and spiral structures. The incorporation of PLGA and β-TCP in PCL provides a much better bone regeneration performance than pure PCL. The reasons were as follows: (1) PLGA can cause faster degradation of the scaffold, thereby forming pores and allowing the cells to infiltrate and grow; (2) β-TCP can release calcium ions, thereby increasing the mechanical strength and osteogenic potential of the scaffold; (3) hybrid PCL and PLGA can overcome the disadvantages of single use and show excellent cell proliferation and tissue formation. Moreover, in contrast with the cylindrical structure, the spiral structure of PCL/PLGA/β-TCP exhibits significantly enhanced cell proliferation and differentiation because of its open architecture, leading to better medium inflow and deeper cell penetration into the scaffold. In summary, this investigation demonstrates that PCL/PLGA/β-TCP scaffold in a spiral shape has great potential as a bone substitute for bone tissue regeneration.

## Data Availability

The original contributions presented in the study are included in the article/Supplementary Material, further inquiries can be directed to the corresponding author.

## Supplementary Materials

The following supporting information can be downloaded at: https://www.mdpi.com/article/10.3390/bioengineering11070732/s1, Figure S1: Photo of three different types of scaffold: PCL cylinder (left), PPBC (middle), and PPBS (right).

## References

[1] Dahlin C., Linde A., Gottlow J., Nyman S. (1988). Healing of bone defects by guided tissue regeneration. Plast. Reconstr. Surg..

[2] Quarto R., Mastrogiacomo M., Cancedda R., Kutepov S.M., Mukhachev V., Lavroukov A., Kon E., Marcacci M. (2001). Repair of Large Bone Defects with the Use of Autologous Bone Marrow Stromal Cells. N. Engl. J. Med..

[3] Salkeld S.L., Patron L.P., Barrack R.L., Cook S.D. (2001). The Effect of Osteogenic Protein-1 on the Healing of Segmental Bone Defects Treated with Autograft or Allograft Bone. JBJS.

[4] Weiland A.J., Moore J.R., Daniel R.K. (1983). Vascularized bone autografts. Experience with 41 cases. Clin. Orthop. Relat. Res..

[5] Wang W., Yeung K.W.K. (2017). Bone grafts and biomaterials substitutes for bone defect repair: A review. Bioact. Mater..

[6] Ai J., Ebrahimi S., Khoshzaban A., Jafarzadeh Kashi T.S., Mehrabani D. (2012). Tissue engineering using human mineralized bone xenograft and bone marrow mesenchymal stem cells allograft in healing of tibial fracture of experimental rabbit model. Iran. Red. Crescent Med. J..

[7] Ghazavi M.T., Stockley I., Yee G., Davis A., Gross A.E. (1997). Reconstruction of Massive Bone Defects with Allograft in Revision Total Knee Arthroplasty. JBJS.

[8] Liu X., Ma P.X. (2004). Polymeric Scaffolds for Bone Tissue Engineering. Ann. Biomed. Eng..

[9] Stratton S., Shelke N.B., Hoshino K., Rudraiah S., Kumbar S.G. (2016). Bioactive polymeric scaffolds for tissue engineering. Bioact. Mater..

[10] Dwivedi R., Kumar S., Pandey R., Mahajan A., Nandana D., Katti D.S., Mehrotra D. (2020). Polycaprolactone as biomaterial for bone scaffolds: Review of literature. J. Oral. Biol. Craniofacial Res..

[11] Ronca D., Langella F., Chierchia M., D’Amora U., Russo T., Domingos M., Gloria A., Bartolo P., Ambrosio L. (2016). Bone Tissue Engineering: 3D PCL-based Nanocomposite Scaffolds with Tailored Properties. Procedia CIRP.

[12] Sheikh Z., Najeeb S., Khurshid Z., Verma V., Rashid H., Glogauer M. (2015). Biodegradable Materials for Bone Repair and Tissue Engineering Applications. Materials.

[13] Fuchs A., Youssef A., Seher A., Hochleitner G., Dalton P.D., Hartmann S., Brands R.C., Müller-Richter U.D.A., Linz C. (2019). Medical-grade polycaprolactone scaffolds made by melt electrospinning writing for oral bone regeneration—A pilot study in vitro. BMC Oral Health.

[14] Makadia H.K., Siegel S.J. (2011). Poly Lactic-co-Glycolic Acid (PLGA) as Biodegradable Controlled Drug Delivery Carrier. Polymers.

[15] Alizadeh-Osgouei M., Li Y., Wen C. (2019). A comprehensive review of biodegradable synthetic polymer-ceramic composites and their manufacture for biomedical applications. Bioact. Mater..

[16] Morgan S.M., Tilley S., Perera S., Ellis M.J., Kanczler J., Chaudhuri J.B., Oreffo R.O. (2007). Expansion of human bone marrow stromal cells on poly-(DL-lactide-co-glycolide) (PDL LGA) hollow fibres designed for use in skeletal tissue engineering. Biomaterials.

[17] Park S.H., Park D.S., Shin J.W., Kang Y.G., Kim H.K., Yoon T.R., Shin J.-W. (2012). Scaffolds for bone tissue engineering fabricated from two different materials by the rapid prototyping technique: PCL versus PLGA. J. Mater. Sci. Mater. Med..

[18] LeGeros R.Z., Lin S., Rohanizadeh R., Mijares D., LeGeros J.P. (2003). Biphasic calcium phosphate bioceramics: Preparation, properties and applications. J. Mater. Sci. Mater. Med..

[19] Mastrogiacomo M., Scaglione S., Martinetti R., Dolcini L., Beltrame F., Cancedda R., Quarto R. (2006). Role of scaffold internal structure on in vivo bone formation in macroporous calcium phosphate bioceramics. Biomaterials.

[20] Yuan H., Kurashina K., de Bruijn J.D., Li Y., de Groot K., Zhang X. (1999). A preliminary study on osteoinduction of two kinds of calcium phosphate ceramics. Biomaterials.

[21] Horch H.H., Sader R., Pautke C., Neff A., Deppe H., Kolk A. (2006). Synthetic, pure-phase beta-tricalcium phosphate ceramic granules (Cerasorb^®^) for bone regeneration in the reconstructive surgery of the jaws. Int. J. Oral Maxillofac. Surg..

[22] Walsh W.R., Vizesi F., Michael D., Auld J., Langdown A., Oliver R., Yu Y., Irie H., Bruce W. (2008). Beta-TCP bone graft substitutes in a bilateral rabbit tibial defect model. Biomaterials.

[23] Getter L., Bhaskar S.N., Cutright D.E., Perez B., Brady J.M., Driskell T.D., O’Hara M.J. (1972). Three biodegradable calcium phosphate slurry implants in bone. J. Oral Surg..

[24] Hettich G., Schierjott R.A., Epple M., Gbureck U., Heinemann S., Mozaffari-Jovein H., Grupp T.M. (2019). Calcium Phosphate Bone Graft Substitutes with High Mechanical Load Capacity and High Degree of Interconnecting Porosity. Materials.

[25] Perche F., Torchilin V.P. (2012). Cancer cell spheroids as a model to evaluate chemotherapy protocols. Cancer Biol. Ther..

[26] Kikuchi M., Koyama Y., Yamada T., Imamura Y., Okada T., Shirahama N., Akita K., Takakuda K., Tanaka J. (2004). Development of guided bone regeneration membrane composed of beta-tricalcium phosphate and poly (L-lactide-co-glycolide-co-epsilon-caprolactone) composites. Biomaterials.

[27] Lam C.X.F., Olkowski R., Swieszkowski W., Tan K.C., Gibson I., Hutmacher D.W. (2008). Mechanical and in vitro evaluations of composite PLDLLA/TCP scaffolds for bone engineering. Virtual Phys. Prototyp..

[28] Kumar A., Zhang Y., Terracciano A., Zhao X., Su T.-L., Kalyon D.M., Katebifar S., Kumbar S.G., Yu X. (2019). Load-bearing biodegradable polycaprolactone-poly (lactic-co-glycolic acid)- beta tri-calcium phosphate scaffolds for bone tissue regeneration. Polym. Adv. Technol..

[29] Jiang H., Zuo Y., Zou Q., Wang H., Du J., Li Y., Yang X. (2013). Biomimetic spiral-cylindrical scaffold based on hybrid chitosan/cellulose/nano-hydroxyapatite membrane for bone regeneration. ACS Appl. Mater. Interfaces.

[30] Zhou S., Zheng T., Chen Y., Zhang J., Li L., Lu F., Zhu J.J. (2014). Toward therapeutic effects evaluation of chronic myeloid leukemia drug: Electrochemical platform for caspase-3 activity sensing. Biosens. Bioelectron..

[31] Wang J., Yu X. (2010). Preparation, characterization and in vitro analysis of novel structured nanofibrous scaffolds for bone tissue engineering. Acta Biomater..

[32] Zhang J., Zhao S., Zhu Y., Huang Y., Zhu M., Tao C., Zhang C. (2014). Three-dimensional printing of strontium-containing mesoporous bioactive glass scaffolds for bone regeneration. Acta Biomater..

[33] Junka R., Quevada K., Yu X. (2020). Acellular polycaprolactone scaffolds laden with fibroblast/endothelial cell-derived extracellular matrix for bone regeneration. J. Biomed. Mater. Res. A.

[34] Manoukian O.S., Aravamudhan A., Lee P., Arul M.R., Yu X., Rudraiah S., Kumbar S.G. (2018). Spiral Layer-by-Layer Micro-Nanostructured Scaffolds for Bone Tissue Engineering. ACS Biomater. Sci. Eng..

[35] Won J.Y., Park C.Y., Bae J.H., Ahn G., Kim C., Lim D.H., Cho D.W., Yun W.S., Shim J.H., Huh J.B. (2016). Evaluation of 3D printed PCL/PLGA/beta-TCP versus collagen membranes for guided bone regeneration in a beagle implant model. Biomed. Mater..

[36] Thi Hiep N., Chan Khon H., Dai Hai N., Byong-Taek L., Van Toi V., Thanh Hung L. (2017). Biocompatibility of PCL/PLGA-BCP porous scaffold for bone tissue engineering applications. J. Biomater. Science. Polym. Ed..

