# Supplementary material for: Fabrication and Evaluation of PCL/PLGA/β-TCP Spiral-Structured Scaffolds for Bone Tissue Engineering
Source: Bioengineering (Basel). 2024 Jul 19;11(7):732. doi: 10.3390/bioengineering11070732 (PMC11274088; doi:10.3390/bioengineering11070732)
Supplement: Supplementary file 1 [file bioengineering-11-00732-s001.zip › bioengineering-3095895-supplementary.pdf]

Fabrication and Evaluation of PCL/PLGA/β-TCP Spiral-Structured Scaffolds for Bone Tissue Engineering

Weiwei Wang [1,†], Xiaqing Zhou [1,†], Haoyu Wang [1], Gan Zhou [2] and Xiaojun Yu [1,*]

[1] Department of Biomedical Engineering, Charles V. Schaefer School of Engineering and Sciences, Stevens Institute of Technology, Hoboken, NJ 07030, USA; wwang19@stevens.edu (W.W.); xzhou12@stevens.edu (X.Z.); hwang40@stevens.edu (H.W.)
[2] Department of Chemistry and Chemical Biology, Charles V. Schaefer School of Engineering and Sciences, Stevens Institute of Technology, Hoboken, NJ 07030, USA
* Correspondence: xyu@stevens.edu; Tel.: +1-201-216-5256
† These authors contributed equally to this work.

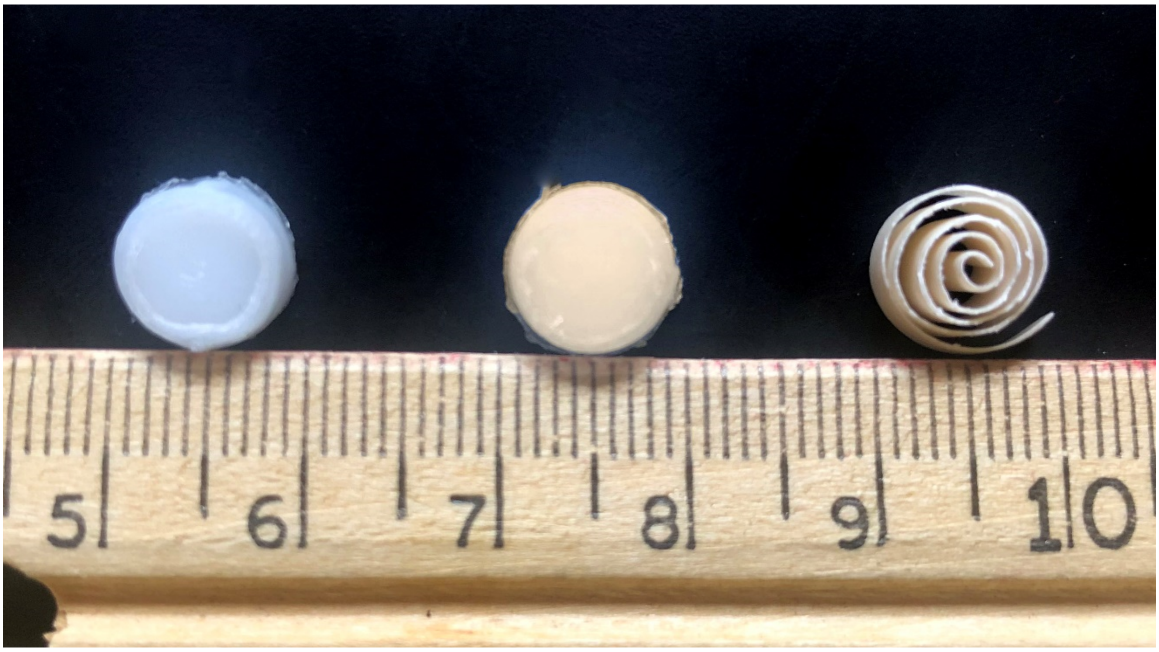

Figure S1. Photo of three different types of scaffold: PCL cylinder (left), PPBC (middle), and PPBS (right).
